# Rev. M. J. Savage on Occultism

**Published:** 1890-02

**Authors:** 


### REV. M. J. SAVAGE ON OCCULTISM.

We extract the following from Rev. M. J. Savage's essay in the December *Forum :*

Long study has driven me to the conclusion that, in a universe the size of this, a modest scientific man will hesitate about declaring as to what is or what is not impossible. The world is perhaps a little too free with its theories as to what can happen and what cannot happen. Not long ago, a workman in a New York factory came to the overseer with a strange story as to the behavior of the steam in a certain part of the works. The overseer, who had made steam his life-long study, declared that the thing was impossible; steam could not act in that way. But investigation proved that the "impossible" was taking place; and the result was a new invention, more knowledge of steam, and an increase in the modesty of the overseer. It is only the traditional court pettifogger who any longer "denies the fact." If it be a fact, then room must be made for it somewhere, however long the explanation of it may have to wait.

I have always tried, then, to see if I could find any facts. I have a horror of being fooled. I have studied sleight-of-hand, and tried to find out the limits and possibilities of trickery. I have, in all directions, wanted the truth and only the truth. I hold that the "scientific method" is the only method of knowledge, and that it can be applied successfully to anything that is real, and with which we really come in contact. I may *hope* a thousand things; I may *believe* that many things are probable; but I have never claimed *to know* anything that could not be demonstrated as true.

In my investigations I have ruthlessly set aside everything that has seemed to occur where the conditions were such that I could not feel sure of my facts. And when I have had the surest grip on a fact, in reasoning upon it I have rigidly tried to explain it in accordance with known laws and forces. It is only when all my knowledge of accepted theories and forces failed to help me to a solution, that I have set the fact aside until some wiser man could tell me what it meant. A study like this, extending over a period of at least a dozen years, has left me what I am to-day. I am in possession of quite a large body of apparent facts that I do not know what to do with. The generally-recognized scientific order of the world has no place for them; I therefore bring them into the open air of the *Forum* to see if any one is wise enough to tell me what they mean. Have they any bearing on the nature and destiny of man? Do they require for explanation the agency of invisible intelligences? Or can they be referred to the working of embodied minds?

That certain things to me inexplicable have occurred, I believe. The negative opinion of some one with whom no such things have occurred, will not satisfy me. Some of those who know the least about such matters will doubtless inform me that I have been deluded, and that my supposed facts are not facts at all. But so long as they do not know the care I have taken, nor the circumstances, and are ignorant of how many times I have repeated the same experiment, this proposed explanation will hardly satisfy me. Neither will it be quite enough to tell me how a similar thing may be done under other conditions. I know all this already, but this knowledge has no bearing on my particular series of facts.

After so much preliminary—none of which, under the circumstances, seems to me uncalled for—I am ready to submit some specimens of those things that constitute my problem. They can be only specimens, for a detailed account of even half of those I have laid by would stretch to the limits of a book.

Though all that has ever been claimed as true, under the general heads of hypnotism, clairvoyance, clairaudience and telepathy, should be proved to be true beyond all question, it is of course apparent that all of them together would still fall far short of proving the spiritualistic claim. For this claim is nothing less than that those we call dead are still alive, and that, at certain times and under certain conditions, they both can and do communicate with persons still in the ordinary body.

And yet, as the very first point in my problem, I wish to submit a case that I suppose falls under the head of telepathy. Out of many I choose this, for the following reasons: It is unquestionably true. Names, dates and all details are accessible. The distance across which the line of communication stretched was enormous. The fact was not expected, and could not have been anticipated. No ordinary method of communication, not even the telegraph, was possible. It is not different in kind from a thousand others; but, like a taller mountain among its fellows, it stands out with peculiar distinctness as a remarkable specimen of its kind.

A merchant ship, bound for New York, was on her homeward voyage. She was in the Indian Ocean. The captain was engaged to be married to a lady living in New England. One day, early in the afternoon, he came, pale and excited, to one of his mates and exclaimed: "Tom, Kate has just died! I have seen her die!" The mate looked at him in amazement, not knowing what to think of such talk. But the captain went on and described the whole scene—the room, her appearance, how she died, and all the circumstances. So real was it to him, and such was the effect on him of his grief, that for two or three weeks he was carefully watched lest he should do violence to himself. It was more than 150 days before the ship reached her harbor. During all this time no news was received from home. But when at last the ship arrived in New York, it was found that Kate did die at the time and under the circumstances seen and described by the captain off the coast of India.

This is only one case out of hundreds. What does it mean? Coincidence? Just happened so? This might be said of one case; but a hundred of such coincidences become inexplicable. Did some invisible intelligence convey the news? Did he really see her? Or did she, in that hour, reach out with such a longing that she touched him half-way round the world?

Now, thougn this may fall short of the spiritualistic claim, does it not suggest something strange and generally unrecognized as to the nature and power of mind? If mind can, under any conditions, or however rarely, assert such a semi-independence of the body and of the ordinary methods of communication, may it not be able to go alone? I do not say or think that such a supposition is proved by a case like this; but is it not at least suggested? When the Second Adventist told Emerson that the world was coming to an end, he calmly replied: "Well, I think I can get along without it." Do not cases like the above at least start the surmise as to whether these souls of ours are not such as to be able to "g t along without it."

From this Mr. Savage goes on to narrate a number of instances of what are usually termed spirit phenomena, including communion with invisible intelligences, the moving of ponderous bodies, etc., etc., quite as startling as thos which the most ardent spiritualist insists upon as evidencing a great truth.

